# Development and Characterization of a Natural Antioxidant Additive in Powder Based on Polyphenols Extracted from Agro-Industrial Wastes (Walnut Green Husk): Effect of Chickpea Protein Concentration as an Encapsulating Agent during Storage

**DOI:** 10.3390/polym16060777

**Published:** 2024-03-12

**Authors:** Daniela Soto-Madrid, Florencia Arrau, Rommy N. Zúñiga, Marlén Gutiérrez-Cutiño, Silvia Matiacevich

**Affiliations:** 1 Food Properties Research Group (INPROAL), Department of Food Science and Technology, Technological Faculty, Universidad de Santiago de Chile, Obispo Umaña 050, Estación Central, Santiago 9170022, Chile; daniela.sotom@usach.cl (D.S.-M.); florencia.arrau@usach.cl (F.A.); 2 Department of Biotechnology, Universidad Tecnológica Metropolitana, Las Palmeras 3360, Ñuñoa, Santiago 8330381, Chile; rommy.zuniga@utem.cl; 3 Molecular Magnetism & Molecular Materials Laboratory (LM4), Department of Chemistry of Materials, Chemistry and Biology Faculty, Universidad de Santiago de Chile, Avenida Libertador Bernardo O’Higgins 3363, Estación Central, Santiago 9170022, Chile; marlen.gutierrez@usach.cl; 4 Center for the Development of Nanoscience and Nanotechnology (CEDENNA), Santiago 9170022, Chile

**Keywords:** storage stability, vegetal protein, by-products, encapsulation, physicochemical properties

## Abstract

Developing a powder-form natural antioxidant additive involves utilizing polyphenols extracted from agro-industrial wastes (walnut green husk). This research explores chickpea proteins (CPP) as an emergent encapsulating agent to enhance the stability and shelf life of the antioxidant additive. This study aims to develop a natural antioxidant powder additive based on polyphenols obtained from walnut green husks encapsulated by chickpea protein (5%, 7.5%, and 10% *w*/*v*) to evaluate their effect under storage at relative humidities (33 and 75% RH). The physicochemical and structural properties analysis indicated that better results were obtained by increasing the protein concentration. This demonstrates the protective effect of CPP on the phenolic compounds and that it is potentially non-toxic. The results suggest that the optimal conditions for storing the antioxidant powder, focusing on antioxidant activity and powder color, involve low relative humidities (33%) and high protein concentration (10%). This research will contribute to demonstrating chickpea protein as an emerging encapsulating agent and the importance of the cytotoxic analysis of extracts obtained from agroindustrial wastes.

## 1. Introduction

There is constant concern about managing agro-industrial wastes. In Chile, the horticultural sector generates approximately 42.5 million tons of waste per year, of which about a million correspond to fruit peels and husks [1] One solution to this problem is to apply processes that reduce waste generation and/or minimize it through its use in the production of secondary goods, known as the circular economy [2].

In addition, consumers worldwide tend to prefer food products containing natural additives, driving the industry to develop these products and ingredients [3]. In this sense, it has been reported that the main active compounds present in various agro-industrial wastes (such as fruit husks or peels) are polyphenols. They have interesting biological and active properties, such as antioxidant and antimicrobial properties [4,5]. Hence, polyphenols could be used as a basis for the development of natural additives that replace synthetic ones. Moreover, according to the market research report from Grand View Research, Inc., the global market size for polyphenols was 1.28 billion USD in 2018, and the annual growth rate for the polyphenol market is expected to reach 7.2% from 2019 to 2025 [6].

Chile occupies the second place among the leading walnut exporters and generates a large amount of waste during the harvest that corresponds mainly to the green husk (exocarp and mesocarp), equivalent to approximately 20% of the total production [7]. This husk has a few uses as a fertilizer due to its organic matter, wood dye, and a replacement for Chinese ink [8,9]. Several studies have shown that green walnut husks are rich in active compounds, mainly polyphenolic compounds with antimicrobial and antioxidant capacities [8,9,10,11]. Therefore, the walnut green husks represent a natural source of active compounds (polyphenols) and a material for extracting these compounds for their potential use as natural additives.

However, the stability of polyphenols is affected by oxygen, light, heat, and water, challenging their incorporation into foods [12]. A possible solution to the instability of polyphenols is the use of an encapsulation technique since it protects, masks, and retains the properties of the active compound [13]. Several materials are used as encapsulating agents, including proteins of animal origin, vegetable proteins, and carbohydrates [14]. A material used as an encapsulating agent should meet several characteristics: low viscosity and hygroscopicity, high solubility in water, absence of odor and taste, ability to form films, and low cost [15]. At the same time, proteins from plant sources showed other advantages, such as biocompatibility, biodegradability, and good amphiphilic and technofunctional properties (such as water solubility, emulsifying, and foaming capacity) [16,17]. Various studies of proteins from plant sources as the encapsulating agent material of active compounds have been reported. For example, soy protein, wheat proteins, zein or prolamin from corn, barley proteins, and other vegetable proteins with high nutritional value from legumes such as lentils, peas, rice, beans, sunflower, and chickpea [16,18,19,20].

Chickpea proteins are emerging biopolymers to be used as an encapsulating agent of drug carriers [20]. They are the third most abundant legume crop globally, with a high protein content (14.9–24.6%) [21] and higher bioavailability than other legumes [22]. Therefore, they could be an alternative to replace animal proteins and protect polyphenolic compounds to develop a plant-based food additive.

It has been reported that phenolic compounds can interact with proteins through non-covalent bonds (hydrophobic interactions and hydrogen bonds), which are generated spontaneously in most food systems. It has been described that the interaction positively influences the sensory, functional, and antioxidant properties of food products [23,24]. For that, chickpea proteins could interact with polyphenols, which can help to protect the antioxidant capacity of phenolic compounds extracted from the walnut green husk and be used to develop new natural food additives. However, food additives must be stable during storage at different relative humidities, which could affect their effectiveness in food matrices. Hence, the objective of this work was to develop a natural antioxidant powder additive based on polyphenols obtained from the walnut green husk (waste from the Chilean agroindustry) and encapsulated using chickpea protein to evaluate their effect under storage at two relative humidities. Moreover, the polyphenolic extract’s identification and cytotoxicity was also evaluated because the walnut green husk was obtained from a traditional Chilean agricultural crop.

## 2. Materials Methods

### 2.1. Samples

The green walnut open husks were obtained from a walnut tree cultivation (*Juglans regia* L.), Chandler variety, in April 2021 in Cuncumen, Province of San Antonio, V Region, Chile. Random sampling was carried out following the methodology of Soto-Madrid et al. [9]. Once the walnut green husks were collected, they were dried in a forced air oven (Zenithlab, DHG-9053 A, Changzhou, China) at 40 °C for 48 h. Then, dried husks were ground in Thermomix equipment (Vorkwerk, Wuppertal, Germany) and stored at room temperature in glass bottles covered with aluminum foil to protect the samples from light.

The chickpea protein (CPP) used as encapsulating material was extracted from commercial chickpea flour (Extrumol, Santiago, Chile) according to the methodology of Soto-Madrid et al. [25]. Briefly, the chickpea protein fraction was obtained by dispersing the defatted flour at alkaline pH (pH = 11.5) and via subsequent isoelectric precipitation (pH = 4.5). Subsequently, the protein was washed with purified water and neutralized to pH 7. Finally, the protein obtained was freeze-dried (Virtis SP Scientific, Benchtop Pro 9L ES-55, Warminster, PA, USA).

### 2.2. Walnut Green Husk Characterization 

Proximal analysis of the walnut green husk and their extracts was performed. Moisture content, proteins, lipids, carbohydrates, ashes, crude fiber, and non-nitrogen extracts were determined according to methods of the Official Association of Analytical Chemistry [26]. 

### 2.3. Extraction of Phenolic Compounds from the Walnut Green Husk

The extraction of phenolic compounds was carried out through ultrasound-assisted extraction (UAE) (Sonics Materials, VCX 500, Newtown, CT, USA) using ethanol–purified water mixture (75:25) as a solvent, with a solid–solvent ratio of 1:25 (*w*:*v*) according to the methodology described by Soto-Madrid et al. [9]. Subsequently, the extract was filtered using a vacuum pump (Rocker, model 300 C, Kaohsiung, Taiwan) and the Whatman paper (N°1). The ethanol was evaporated in a rotary evaporator (Buchi R-100, Flawil, Switzerland) at a temperature of 40 °C. Finally, the extract obtained was stored in a 200 mL amber bottle and refrigerated until further analysis.

#### 2.3.1. Quantification of Total Polyphenol Content and Antioxidant Capacity

Total phenolic content (TPC) was determined using the Folin–Ciocalteu method with some modifications [27]. Briefly, 0.1 mL of sample was added to a 10 mL volumetric flask with 4.9 mL of distilled water and 0.5 mL of Folin–Ciocalteu reagent (Merck, Darmstadt, Germany), followed by 1.7 mL of Na_2_CO_3_ (20% *w*/*v*, Merck, Darmstadt, Germany) addition. Then, distilled water was added until it reached 10 mL. The reactive mixture was allowed to stand in darkness for 2 h as an indicator of TPC, and the formation of a blue color was quantified at 740 nm using a spectrophotometer (Shimadzu UVmini-1240, Kyoto, Japan). Gallic acid (Merck, Darmstadt, Germany) was used to construct the standard curve (0.1 to 0.8 mg/mL). Results were expressed as milligrams of gallic acid equivalents/g sample dry weight (mgGAE/g dw). All assays were performed in triplicate.

The antioxidant capacity was determined by scavenging 2,2-diphenyl-1 picrylhydrazyl (DPPH), according to the method reported by Brand-Williams et al. [28], with some modifications. Briefly, 50 μL of diluted concentrations of the walnut green husk extract and the powders developed (reconstituted at 1% *w*/*v*) were mixed with 2950 μL of a methanolic solution containing the DPPH radical (concentration 80 mg/L) (Sigma-Aldrich, St. Louis, MI, USA). The mixture was stirred and left in the dark for 30 min, and subsequently, its absorbance at 517 nm was measured using a spectrophotometer (Shimadzu, UVmini-1240, Kyoto, Japan). The standard curve was constructed using Trolox (Sigma-Aldrich, St. Louis, MI, USA) (0 to 1600 μM), and the results were expressed as mg Trolox/g sample dry weight (mg Trolox/g dw). All assays were performed in triplicate.

#### 2.3.2. Identification of Phenolic Compounds

The identification of the phenolic compounds present in the walnut green husk extract was carried out in a performance liquid chromatography system coupled to mass spectrometry (UPLC-QTOF-ESI-MS, Waters Xevo G2-XS QTof/Tof, Waters, Milford, MA, USA). Chromatographic separation was conducted on an ACQUITY UPLC^®^ Hss T3 column (2.1 × 100 mm, 1.8 μm particle size). The mobile phase gradient followed the following sequence: at time 0 (97% A and 3% B), at 30 min (3% A and 97% B), and from 35 to 40 min (97% A and 3% B).

The mass spectrometer was operated in both positive and negative ion modes. The identification method was carried out using the Progenesis QI MetaScope v2.3 software (Waters, Milford, MA, USA), and the search parameters were established through the HMDB library. The precursor ion and the fragments used an error tolerance of 10 ppm. All compounds found with adduct formation in M-H and M+H modes are reported as results. A mass range of 100 to 1000 Da was taken into account.

### 2.4. Cell Viability Assay Using Vero Cells

Resazurin was used to quantify the viability of Vero cells. This compound fluoresces when cells metabolize resazurin and reduce it to resofurin. The amount of resofurin produced is proportional to the cell metabolic activity and can therefore be used to evaluate cell viability, where high fluorescence indicates high cell viability. Briefly, 5000 Vero cells were seeded per well in 96-well microplates. Then, 20 μL of 0.5 mg/mL resazurin solution in PBS (Phosphate Buffered Saline) was added per well and incubated at 37 °C for 4 h in a humidified environment with a concentration of 5% CO_2_. The wells were then analyzed by fluorescence (Tecan, Infinite 200 Pro reader, Mennedorf, Switzerland), with an excitation wavelength of 560 nm and an emission wavelength of 590 nm. The data were analyzed with the statistical software GraphPad Prism 9.5.1 to determine the cytotoxic concentration of each extract that reduces viability by 50% (CC_50_). 

The Vero cell line was obtained from Dr. Cesar Echeverria from the University of Antofagasta, Chile.

### 2.5. Development of the Active Antioxidant Additive

For the assays, chickpea protein was mixed at three concentrations (5, 7.5, and 10% *w*/*v* in 40 mL) with the walnut green husk extract (20 mL) at room temperature for 3 h with stirring (490 rpm) and under dark conditions until the complete dispersion of the protein. This methodology favored complete protein dispersion and the non-covalent interaction between protein and polyphenols [29]. Afterward, the samples were freeze-dried at −18 °C and placed in the freeze-dryer chamber collector at −60 °C with a shelf at 30 °C under a pressure of 0.05 bar for 72 h (Ilshin FD5508, Siheung-si, Republic of Korea). Three samples were obtained: FDP 5%, corresponding to the additive that contains CPP at 5% *w*/*v*; FDP 7.5%, corresponding to the additive with 7.5% *w*/*v* of CPP; and FDP 10%, corresponding to the additive that contains 10% *w*/*v* CPP.

### 2.6. Physicochemical Characterization of Additives in Powders

#### 2.6.1. Encapsulation Efficiency (E.E. %)

The encapsulation efficiency was calculated by the total polyphenol content of the powder additive and the extract using the following equation:

(1) $$E.E.\left(\%\right)=\frac{TPCE-TPCP}{TPCP}*100\%,$$

where TPCE corresponds to the total polyphenol content of the sample before freeze-drying, and TPCP corresponds to the polyphenol content of the powder additive.

#### 2.6.2. Drying Process Yield (DY %)

The total solids of the extract with the encapsulating agent (SST, soluble solids/100 mL of extract) were measured via refractometry (RHB-32 ATC, YHEquipment, Shenzen, China) before drying. Once the sample was dried, the powders were weighed on an analytical balance (HR-120, A&D Co., Tokyo, Japan). With the weights, the extraction yield (DY%) was calculated using the following Equation (2) according to the conditions described by Fenoglio et al. [30]:

(2) $$DY\left(\%\right)=\frac{Total\;solid\;after\;freeze-drying}{Initial\;total\;solids}*100\%.$$

#### 2.6.3. Moisture Content and Water Activity

The moisture content of the dry powders was determined gravimetrically by the difference in mass before and after drying the samples in an oven (Shel Lab 1410-2E, Capovani Brothers, New York, NY, USA) at 105 °C until constant weight (AOAC, 1998). Results were expressed as dry basis percentage (% db; g water/100 g solids). The water activity (a_W_) of walnut green husk dried and additive powders was determined using a chilled-mirror dew point device (Aqualab, Series 3 TE, Decagon, Washington, DC, USA) at 25 °C (AOAC, 1998).

#### 2.6.4. Color Analysis

The color of the additives was determined through image analysis using a computer vision system (previously calibrated). It consists of a black box with four natural lights D65 (18 W, Phillips, Amsterdam, The Netherlands) and a digital camera (Canon, EOS Rebel XS, Tokyo, Japan) at a distance of 22.5 cm from the sample (camera lens angle and lights at 45°) [31]. Samples were measured as pellets by pressing powders with a Quick Press hand press (Perkin-Elmer, Waltham, MA, USA). The digital color parameters were obtained in the RGB space using the software Adobe Photoshop v7.0 (Adobe Systems Incorporated, 2007), which was subsequently converted to the CIELAB space, in which L* indicates lightness, a* the red-green axis, and b* the blue-yellow axis.

#### 2.6.5. Structural Characterization: SEM and FTIR 

Scanning electron microscopy (SEM) was employed to analyze the microstructure of the additives using a field emission scanning electron microscope (Zeiss, model EVO MA10, Jena, Germany). The samples were affixed to stubs using double-sided adhesive tape, coated with a gold layer, and images were captured with an acceleration voltage of 20 kV.

The chemical groups and bonding arrangement of components present in the samples were determined by Fourier transform infrared–attenuated total reflectance (FTIR-ATR) using an infrared spectrophotometer equipped with an ATR PRO ONE (Jasco FTIR-4600, Easton, MD, USA). Measurements were performed in a spectral range from 4000 to 400 cm^−1^, with a resolution of 4 cm^−1^ and 32 scans per sample.

#### 2.6.6. Isoelectric Point (IEP) 

The isoelectric points (IEP) were determined through Zeta Potential (pZ) measurements (Zetasizer Nano Series, NanoZS90, Malvern Instruments, Malvern, UK). Dilute suspensions of the powder additive (approximately 0.05 g/L) were prepared in 10^−3^ mol/L KCl, and the pH was adjusted using 10^−2^ mol/L HCl or KOH. The IEP was identified as the pH value where pZ equals zero.

### 2.7. Stability at Different Relative Humidities (RH)

The developed additives were evaluated in hermetically sealed desiccators at different relative humidities for two weeks. Saturated solutions of MgCl_2_ and NaCl were used to obtain 33% and 75% RH, respectively [32]. The polyphenol total contents, antioxidant activity, water activity, and color parameters (L*, a*, and b*) were measured at the beginning and end of the analysis.

### 2.8. Statistical Analysis

All experiments were run in triplicate. Data were reported as means with their corresponding standard deviation. ANOVA test was performed at a confidence level of 95% to determine statistical differences using Statgraphics Centurion XVI^®^ software (StatPoint Technologies Inc., Warrenton, VA, USA, Version XVI). Differences between samples were evaluated using multiple range tests, using the least significant differences (LSD) multiple comparison method. The significance of the differences was determined at a 95% confidence level (*p* < 0.05). The linear dependency between two independent variables was obtained by the r-Pearson coefficient using Microsoft Excel v10.

## 3. Results and Discussion

### 3.1. Characterization of the Walnut Green Husk and Extracts

#### 3.1.1. Proximal Analysis 

It is essential to characterize the walnut green husk and the extract obtained from it through proximal analysis to standardize the extraction process (Table 1). The raw material presented an a_W_ value lower than 0.6, ensuring their stability against microbial growth [33] before each extraction.

As expected, due to the ultrasound-assisted extraction process, the extracts’ proximal analyses (Table 1) showed non-detection (ND) of compounds such as lipids, proteins, and fibers, indicating no contamination during the extraction process and no possible interactions with proteins of these compounds that affect the polyphenol protection. Moreover, the extraction efficiency in the extract obtained was 100% from the walnut green husk, so the extraction methodology is validated to obtain non-nitrogenous extracts (N.N.E.). Besides, 83.3% of the total dry sample of N.N.E. was obtained, which comprises soluble compounds such as polyphenols, phenolic acids, and flavonoids since they do not have a group functional based on nitrogen in their structure [13]. However, the ash content (16.7% of the total dry sample) was attributed to soluble minerals of the raw material, which can act as electrolytes and could negatively affect the stability of the protein–polyphenols interaction [35]. Increasing chickpea protein concentrations must be studied to avoid this potential effect.

Independently of this, it is important to note that ultrasound-assisted extraction is a simple, efficient, and sustainable technique [36] that allows for better penetration of solvents, a shorter extraction time, and higher extraction yield of polyphenols, even at lower temperatures compared to other extraction methods of phenolic compounds from plant matrices [37]. 

In parallel, the polyphenol content and its antioxidant capacity were determined via the DPPH method to (i) confirm that the compounds present in the extract (N.N.E.) are polyphenols and (ii) if they maintain their antioxidant activity after the extraction process. The walnut green husk sample harvest in 2021 presented a 36% higher value for TPC and a similar value for antioxidant capacity (202 ± 1.2 mg GAE/g dry sample) compared to the harvest in 2019, previously reported by Soto-Madrid et al. [9]. It could be attributed to differences in the polyphenol type and quantity extracted but with the same activity. However, few compounds were reported with which to compare it. Moreover, the differences could also be due to raw material differences, which may vary according to ripeness stage, environmental factors, and the mode of collection and storage [38]. For that, it is crucial to identify the compounds in the polyphenolic extract and evaluate the efficacy of the extraction process.

#### 3.1.2. Identification of Compounds in the Walnut Green Husk Extract

The compounds identified by UPLC-QTOF-ESI-MS in negative and positive modes, where the mass/charge (*m*/*z*) values were also compared to those reported by Sheng et al. [39], are shown in Table 2. Briefly, 64 compounds were identified, including hydrolyzable tannins, flavonoids, phenolic acids, phenolic glycosides, and quinones. Unexpectedly, herbicides and fungicides were also identified due to the traditional agricultural fields that use these pesticides as a common practice.

Of the total identified compounds (64), 29% corresponded to phenolic acids such as gallic acid, protocatechuic acid, and ferulic acid, which had also been identified by Soto-Madrid et al. [9] (2021) via HPLC-RP. Then, 22% corresponded to flavonoids such as quercetin, quercitrin, catechin, kaempferol, and others; 15% to phenolic glycosides; 4.5% of hydrolyzable tannins; and 3% of quinones. This 73.5% phenolic compounds demonstrated antioxidant activity [13]. However, 3% was attributed to pesticides, which could negatively affect health. Therefore, to use the walnut green husk extract as a base to develop a natural food additive, it is required to evaluate its cytotoxicity through in vitro studies with cell cultures.

#### 3.1.3. Cytotoxicity Evaluation of Walnut Green Husk Extract

The literature has reported that an extract can be considered very toxic with a CC_50_ < 10 μg/mL, moderately toxic with CC_50_ = 11–30 μg/mL, slightly toxic at CC_50_ = 31–50 μg/mL, and potentially non-toxic at CC_50_ > 50 μg/mL [40]. The cell viability assay of this work’s walnut green husk extracts was CC_50_ = 90 ± 9 μg/mL, demonstrating that it is potentially non-toxic and could be used to develop a natural antioxidant additive based on agroindustrial waste. However, it is essential to consider the traces of these compounds for another possible adverse effect, such as potential allergenicity, which was not evaluated and must be labeled and regulated. Considering this result, it is necessary to realize a cell viability study in extracts obtained from wastes due to the growing tendency of waste revalorization in agroindustry, where the use of pesticides is a common agricultural practice.

### 3.2. Development of the Natural Antioxidant Additive

#### 3.2.1. Physicochemical Characterization of the Natural Antioxidant Powder Additive

Different concentrations of chickpea protein (5, 7.5, and 10% *w*/*v*) were studied as encapsulating material of the phenolic compounds to develop the additive. As expected, the freeze-drying showed high values of drying process yield (DY%), close to ~98.6% for all samples, confirming that this process generates low losses in terms of solids recovery. This is a positive aspect since, for example, in the spray drying process, the DY% is low (60–90%) due to losses of solids occurring by their adhesion to the drying chamber [41]. Also, high wall material concentrations are required to protect the active compound at high temperatures [16]. 

Table 3 shows the results of the physicochemical characterization of the powder obtained by freeze-drying. The highest E.E. (%) was obtained for the FDP 7.5% (60 ± 6%) sample. Compared with the literature, spray-drying is a better process by which to obtain a high E.E.% (65–92%) of polyphenol compounds using proteins as encapsulating agents [41]. The encapsulation efficiency differences reported in the literature could be related to the nature of the polyphenolic compounds (i.e., charge, type of compound, chemical structure) and the structure of the wall material, positively or negatively conditioning the polyphenol–polymer interaction since they are the most critical variables to consider for the encapsulation of polyphenols [42]. It considered that the drying technique and the material used as protection affected the retention capacity of compounds within the matrix. For that, selecting the wall material and the drying technique is crucial to balance high drying process yield and encapsulation efficiency to maximize the incorporation and retention of the functional compounds within the encapsulation matrix.

The water activity (a_W_) and moisture content are critical physical parameters of powdered additives since they strongly influence their storage stability and safety. In this sense, all the powders analyzed presented a_W_ lower than 0.2, demonstrating safety and low biochemical kinetic reactions [33]. The FDP 5% sample showed the highest value moisture content (7.800 ± 0.003% db), and it was statistically different (*p* < 0.05) when compared with the FDP 7.5% and 10% samples (approx. 6% db) (Table 3). It could be attributed to the lower concentration of protein to protect the phenolic extract. The ice crystals’ sublimation during freeze-drying generated many small porous and less compact structures, resisting mass transfer and acting as a barrier against sublimation [43]. It results in greater moisture retention and, consequently, higher moisture in the final product.

Table 3 also shows the color parameters considered in the CIELAB L*a*b* space. The lightness parameter (L*~77) showed no statistically significant differences (*p* > 0.05) in the three samples analyzed. This high L* value (scale 0–100) indicates light powders. However, for the a* parameter, the 7.5% sample presented the highest value (5.9 ± 0.5). It was significantly different (*p* < 0.05) compared to the other two samples, which shows a little tendency towards the red color. On the other hand, for the b* parameter, the FDP 10% sample presented the highest value (10.6 ± 0.6). It was statistically different (*p* < 0.05) from the other samples, indicating the tendency towards the yellow color. The changes in a* and b* parameters are attributed to a higher protein concentration, generating greater protection for the brown phenolic extract, which begins to turn yellow, the characteristic color of chickpea protein. Independent of the significant differences in each parameter observed, the visual color and chromaticity diagram (xy scale) indicated light powders with a little tendency to yellow at higher protein concentrations. Furthermore, in the images (Table 3), it can be observed that at high protein concentrations, the compaction of the powder additive increased. Therefore, all the samples analyzed had low water activity, this is favorable for their storage and shelf life and, combined with their light color, would allow for their addition to food matrices.

#### 3.2.2. Structural Characterization of Powders

**Figure 1:**
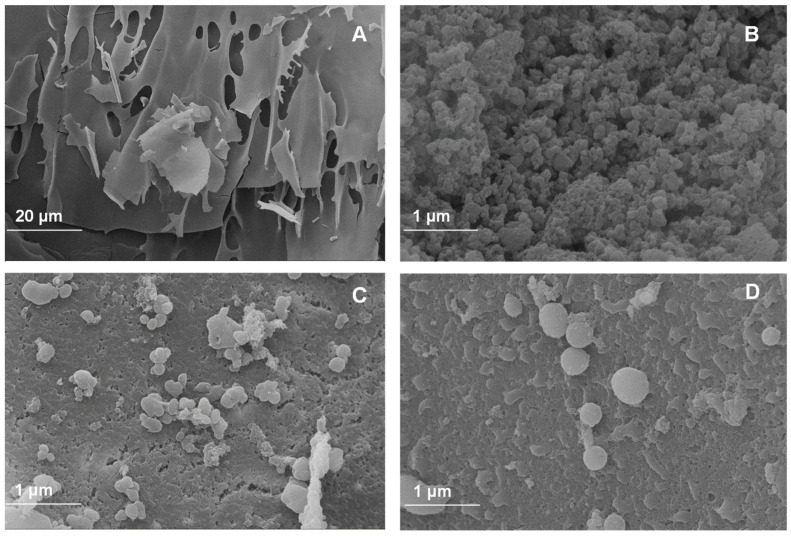
Images of antioxidant powder additives: (**A**) control protein; (**B**) freeze-dried powder additive with 5% chickpea protein; (**C**) freeze-dried powder additive with 7.5% chickpea protein; (**D**) freeze-dried powder additive with 10% chickpea protein.

Scanning electron microscopy (SEM) was performed to evaluate the morphology of the powder additives based on the walnut green husk extract. Micrographs correspond to the CPP control (Figure 1A) used as wall material and to the powder additives with different percentages of CPP (Figure 1B–D). Figure 1A shows an irregular, brittle, and flake-like structure, a common structural characteristic (at 20 μm) of freeze-dried proteins [44,45]. A porous structure with irregular shapes and sizes is evident in the FDP 5% sample (Figure 1B). This sample porosity may result from ice formation in the material during the freeze-drying process. However, as the CPP concentration increases in the development of the powder additive, a more defined, almost spherical morphology is observed with the formation of larger capsules and a decrease in porosity (Figure 1C,D). This is evidence of the encapsulation of phenolic compounds from the walnut green husk extract using concentrations of 7.5 and 10% CPP.

**Figure 2:**
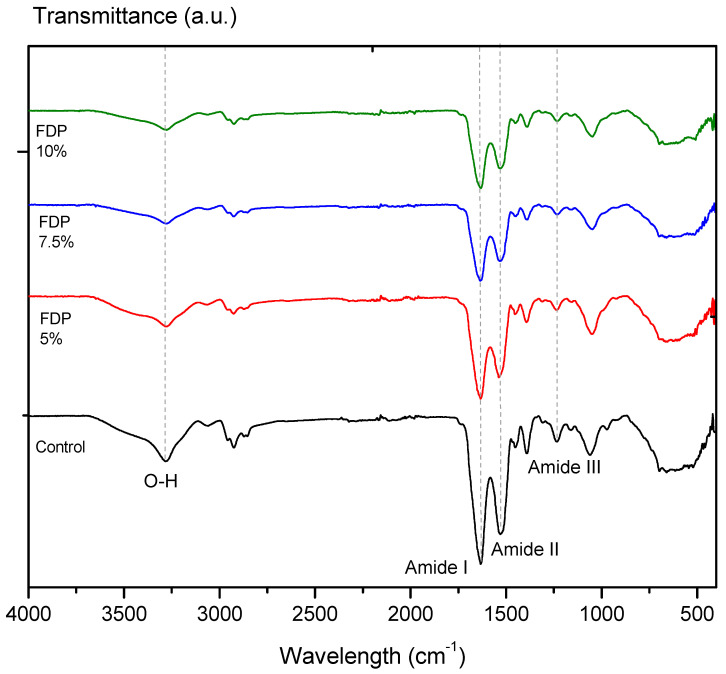
Infrared spectra by Fourier transform of additives in powders: Control: freeze-dried chick-pea protein; FDP 5%: freeze-dried powder additive with 7.5% chickpea protein; FDP 7.5%: freeze-dried powder additive with 7.5% chickpea protein; FDP 10%: freeze-dried powder additive with 10% chickpea protein.

The protective effect of CPP on the active compounds from the walnut green husk extract can be evidenced by a decrease or displacement of the typical signals of the bands measured by FTIR [20]. Figure 2 illustrates the FTIR spectra of the freeze-drying additives and the control sample corresponding to CPP. All FTIR spectra showed a typical absorption band at a wavelength of ~3278 cm^−1^, characteristic of the water’s hydroxyl group (–OH). As a result of encapsulation, the C=O stretching of the amide I band of CPP at ~1633 cm^−1^ has shifted slightly to ~1636 cm^−1^ in the spectrum of the FDP 7.5% sample. In parallel, amide II: N–H bending and C–N stretching of proteins at 1530 cm^−1^ has shifted to 1533 and 1538 cm^−1^ in the FDP 5% and FDP 7.5% samples. In addition, it also identified a band around ~1235 cm^−1^ corresponding to the amide III region (CN stretching, NH bending) [46], which has shifted slightly to 1233 cm^−1^ in the FDP 10% sample. Although the band displacements are between 2–3 cm^−1^, the literature using chickpea protein reported these little changes as component interactions [20]. Moreover, CPP peaks are weakened in intensity due to the encapsulation of phenolic compounds [20].

#### 3.2.3. Zeta Potential (pZ)

**Figure 3:**
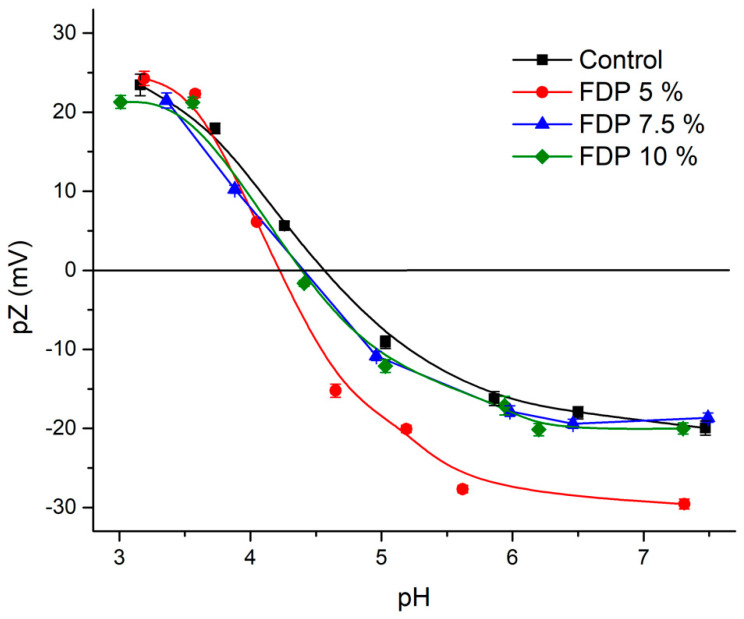
Zeta potential (pZ) as a function of pH for control protein (black), FDP 5% (red), FDP 7.5% (blue), and 10% FDP (green) samples. FDP = freeze-drying powder at different chickpea protein concentrations in % *w*/*v*.

Zeta potential is an important and valuable indicator of particle surface charge, which can be used to predict and control the stability of suspensions [47,48]. Figure 3 shows the control protein’s zeta potential versus pH curve and FDP 5, 7.5, and 10% samples. At pH ~3, all samples present values between |20–25| mV, independent of the protein concentration used. This indicates that they are outside of the flocculation region (|5–15| mV) and near the optimal region |30| mV, evidencing the stability of the powder additives at this pH. So, this indicates potential applications in acid food matrices. Moreover, at pH 6–7, there were differences in the values obtained, where the FDP 5% sample presented the highest value (|30| mV) compared to the other samples (|~20| mV). It can be attributed to the acid-base properties of different radicals charges or functional groups due to the structural characteristics of each flavonoid (present in the walnut green husk extract), which showed a negative charge at pH ~7 [49], contributing to the total surface charge and evidencing that they were not protected (free) in the FDP 5% sample.

As expected, the control exhibits an isoelectric point (IEP) value of 4.55, similar to the 4.5 reported by Soto-Madrid et al. [25], while FDP 7.55% and 10% display consistent IEP at 4.45 with similar behavior. However, FDP 5% exhibits a slightly lower IEP of 4.3. All values agree with Vani and Zayas. [50], Boye et al. [51], and Ma et al. [52]; the authors indicate that most plant proteins have an IP between 4.0 and 5.0. It is important to note that proteins could adsorb charges on their surfaces at their IEP due to the presence of other compounds, such as polyphenols [53]. In this case, the diminution of IEP in the FDP 5% sample can be attributed to negative charges from free phenolic compounds, independent of the total neutral charge at IEP.

### 3.3. Stability of the Antioxidant Additive at Different Relative Humidities (RH)

The stability of antioxidant powder additives depends mainly on their water activity since water can act as a reagent or solvent in different degradation reactions or contribute to microbial growth. Furthermore, the water content of a freeze-drying product depends on the residual moisture left in the product after drying and the water that it can adsorb from the surrounding atmosphere during storage [54]. 

**Figure 4:**
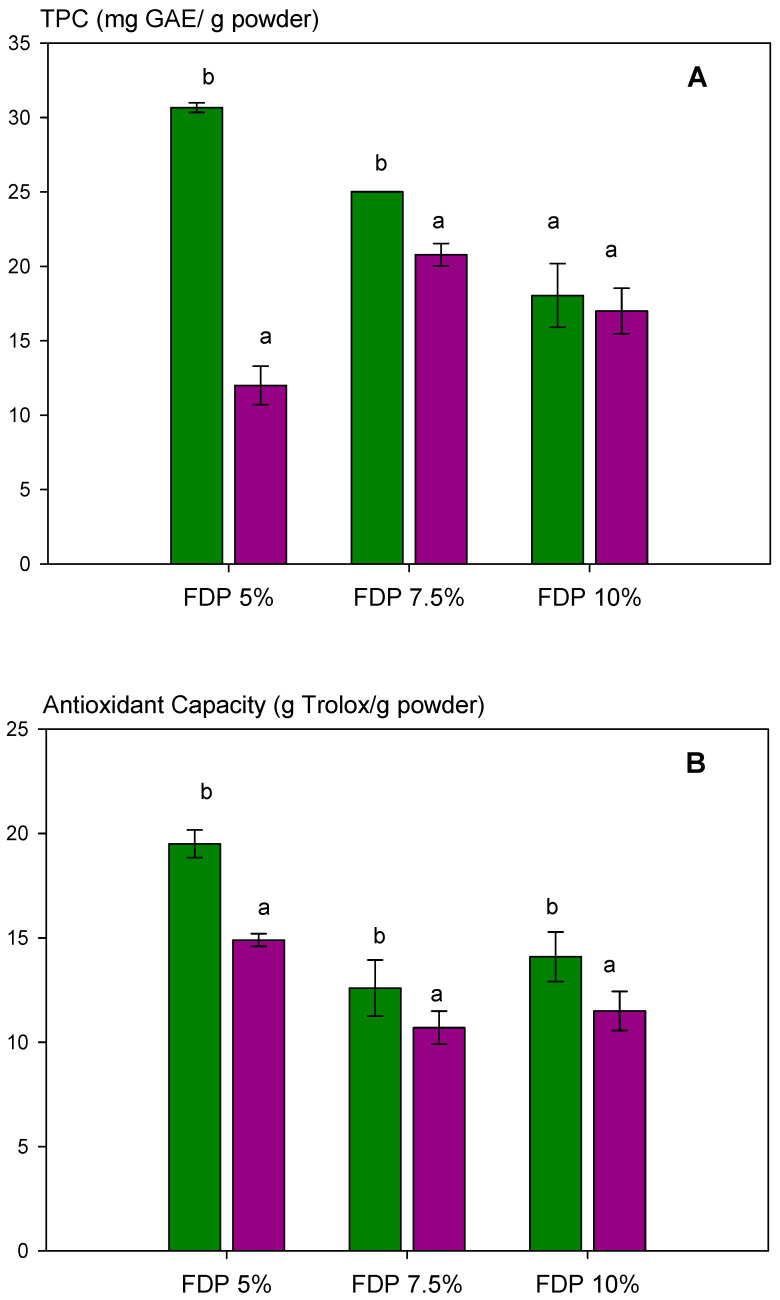
Analysis of the stability of the total polyphenol content and antioxidant activity of powder additives at different relative humidities (RH): (**A**) total polyphenol content (TPC); (**B**) antioxidant capacity of the powders quantified by DPPH radical scavenging activity. Green bars indicate relative humidity 33%, and violet bars indicate a relative humidity 75%. FDP = freeze-drying powder at different chickpea protein concentrations in % *w*/*v*. GAE = gallic acid equivalent. Different letters (a,b) indicate significant differences (*p* < 0.05) between samples.

The effect of two relative humidities (33 and 75% RH) on antioxidant capacity via DPPH and total polyphenol content, TPC, was analyzed in powder additives. The results are shown in Figure 4A (TPC) and Figure 4B (antioxidant capacity). Relative humidity affected TPC, decreasing significantly at 75% RH, dependent on protein concentration (Figure 4A). For the FDP 5% sample, a 60% diminution of TPC was observed; meanwhile, at 7.5%, it was 17%. However, relative humidity did not significantly affect (*p* > 0.05) the TPC when the protein concentration was 10%. This demonstrated the importance of protein concentration in protecting the antioxidant compounds from humidity during storage. The same behavior was observed for antioxidant capacity (Figure 4B). 

Interestingly, TPC and antioxidant capacity diminished as protein concentration increased at 33% RH. It can be attributed to higher protection and protein–polyphenols interaction when increasing the protein concentration, as shown in Figure 2. Moreover, the results above indicated the presence of free polyphenols in the FDP 5% sample, which are corroborated in Figure 3 with higher TPC and antioxidant activity, independent of relative humidity.

**Figure 5:**
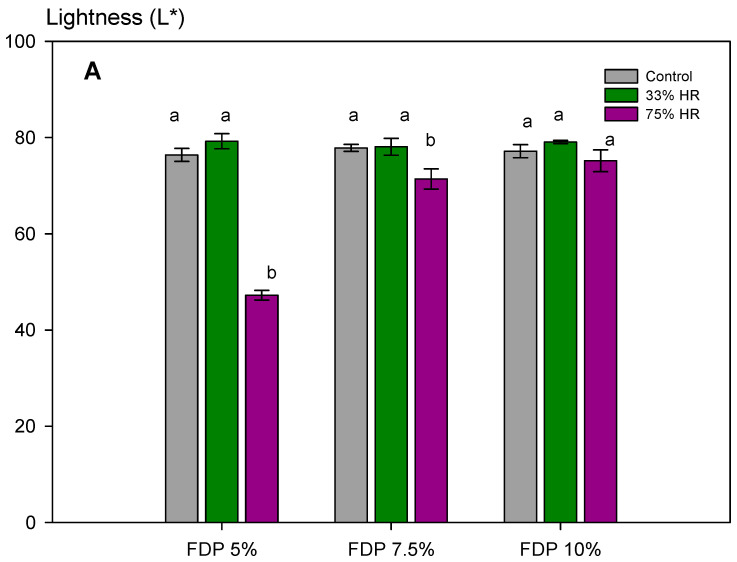

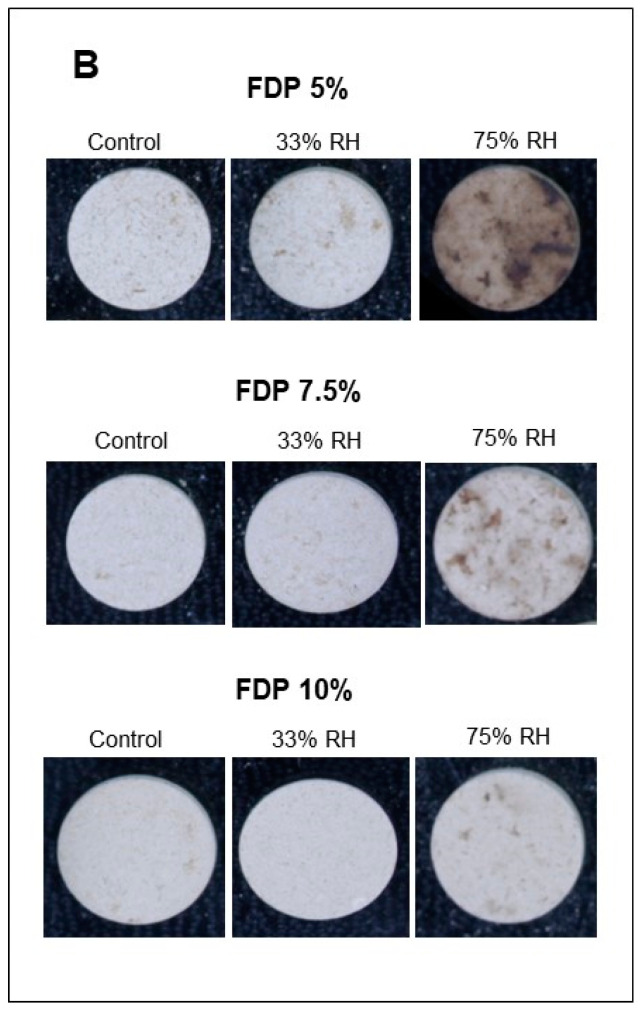
Stability of the additive powders for the lightness parameter (L*) at different relative humidities (RH) and protein concentrations (%). (**A**) Graph of the analysis of the L* parameter for powder additives. (**B**) Images of the powder additives: control, 33, and 75% RH. Green bars indicate relative humidity 33%, and violet bars indicate a relative humidity 75%. FDP = freeze-drying powder at different chickpea protein concentrations in % *w*/*v*. Different letters (a,b) indicate significant differences (*p* < 0.05) between samples.

Furthermore, for the stability of the powders during storage, it is essential to maintain the activity of antioxidant compounds and the powder color. Figure 5 shows the stability of the additive powders for the lightness parameter (L*) at the different relative humidities and the visual changes. The effect of relative humidity in lightness was insignificant at 33% RH, independent of protein concentration. However, at 75% RH, the effect was also dependent on protein concentration, with the lowest loss at 10% of protein concentration (FDP 10%). As expected, considering the above results, the stability of samples at 75% RH was lower, showing a dark powder after storage. For that, a 33% RH confirms the stability of the antioxidant powder additive during storage. The FDP 10% sample maintained the lightness at 33% RH and exhibited the lowest change in color when stored at higher relative humidity.

It is important to note that the higher encapsulation efficiency obtained at 7.5% *w*/*v* of the encapsulating agent is not correlated to a higher TPC and antioxidant capacity during storage conditions, as expected. In this case, it is attributed to oxidized phenolic compounds in the powder surface, which is correlated (r-Pearson = 0.9707) to the a* parameter (Table 3).

Nevertheless, considering the cost of the freeze-dried process, the study highlights the need for further investigations to bolster these findings compared to widely used encapsulation technologies like spray drying. Such comparative analyses will provide a more comprehensive understanding of the relative effectiveness and feasibility of the developed antioxidant powder additives. The ongoing pursuit of knowledge in this area will contribute valuable insights to the field and facilitate informed decision-making for industrial applications.

## 4. Conclusions

The extracted compounds obtained from walnut green husk showed the presence of phenolic acids, flavonoids, hydrolyzable tannins, and quinones, which are responsible for the antioxidant capacity of the extract. Moreover, herbicides and pesticides were also identified. Still, the extract is potentially non-toxic and can be used as a matrix for phenolic extraction to develop natural additives. Chickpea proteins are shown in this study as emerging polymers for encapsulating the phenolic extract from walnut green husk. The FDP 10% sample presented the best values in the physicochemical and structural characterization, demonstrating the protective effect of chickpea protein on the active compounds. Considering only the antioxidant activity and powder color of additives developed at high protein concentrations (10%), the best storage condition for these powders is a low relative humidity (33%) to maintain the antioxidant compounds’ stability. This study demonstrated the importance of storage stability studies for powder-form natural additives. Further studies will require applying this additive to different food matrices and studying its behavior as an antioxidant additive through concentration and sensorial analyses.

## Figures and Tables

**Table 1:** Proximal analysis of the walnut green husk and liquid extract corresponding to the 2021 harvest.

Analysis	Walnut Green Husk (g/100 g Dry Base)	Extract Liquid (g/100 g Wet Base)
Moisture	7.5 ± 0.01	93.9 ± 0.01
Proteins (%Nx5.3 ^1^)	6.98 ± 0.23	* ND
Lipids	1.94 ± 0.11	** ND
Ash	12.6 ± 0.05	1.0 ± 0.06
Crude fiber	20.34 ± 1.19	*** ND
Non-nitrogen extract (N.N.E.)	50.66 ± 1.13	5.0 ± 0.08
Energy (Kcal)	248 ± 4.41	19.9 ± 0.31

* ND: detection limit ≤ 0.39 g/100 g; ** ND: detection limit ≤ 0.52 g/100 g; ***: detection limit ≤ 0.59 g/100 g. ^1^ Conversion factor for nuts [34].

**Table 2:** Compounds identified in the walnut green husk extract by UPLC-QTOF-ESI-MS.

N°	RT (min)	Formula	Measured Mass (*m*/z)	Error (ppm)	Ion Mode	Compound Identification	Reference	Classification
1	1.30	C_11_H_14_NO_7_^+^	271.0687	−4.03	M-H	pyridine N-oxide glucuronide	[a]	Other (aromatic compound)
2	1.55	C_4_H_6_O_6_	133.0138	−6.03	M-H	malic acid	[a]	organic acid
3	2.89	C_13_H_16_O_10_	331.0669	−0.41	M-H	3-glycogallic acid	[a]	Phenolic glycosides
4	3.14	C_7_H_6_O_5_	169.0140	−1.37	M-H	gallic acid	[a]; [b]	Phenolic acid
5	3.64	C_14_H_16_N_2_O_8_	321.0729	0.08	M-H	glutamic acid-betaxanthin	[a]	Vegetal pigment
6	3.86	C_14_H_18_O_10_	345.0835	2.27	M-H	methyl 6-O-galloyl-beta-D-glucopyranoside	[a,b]	Hydrolyzable tannin
7	3.86	C_8_H_8_O_5_	183.0303	2.35	M-H	methyl gallate	[a]	Phenolic compound
8	4.13	C_15_H_20_O_10_	359.1002	5.13	M-H	3-methoxy-4-hydroxyphenylglycol-glucuronide	[a]	Phenolic glycosides
9	4.20	C_9_H_10_O_4_	181.0505	−0.61	M-H	syringaldehyde	[a]; [b]	Aromatic aldehyde
10	4.21	C_8_H_8_O_3_	151.0399	−1.58	M-H	vanillin	[a]; [b]	Phenolic aldehyde
11	4.47	C_13_H_16_O_8_	299.0773	0.29	M-H	4-methylcatechol 1-glucuronide	[a]	Phenolic glycosides
12	4.52	C_7_H_6_O_4_	153.0189	−2.89	M-H	protocatechuic acid	[a]; [b]	Phenolic acid
13	4.62	C_9_H_8_O_4_	179.0356	3.67	M-H	caffeic acid	[a]; [b]	Phenolic acid
14	4.62	C_7_H_12_O_6_	191.0561	0.02	M-H	quinic acid	[a]; [b]	Phenolic acid
15	4.62	C_16_H_18_O_9_	353.0879	0.31	M-H	crypto chlorogenic acid	[a]	Phenolic acid
16	4.69	C_14_H_18_O_9_	329.0885	2.13	M-H	vanillyl glucose	[a]	Hydrolyzable tannin
17	4.93	C_15_H_20_O_10_	359.0985	0.29	M-H	glucosyringic acid	[a]	Phenolic glycosides
18	5.00	C_30_H_26_O_12_	577.1342	−1.56	M-H	procyanidin B8	[a]; [b]	Flavonoid
19	5.02	C_8_H_8_O_4_	169.0497	1.06	M+H	isovanilic acid	[a]; [b]	Phenolic acid
20	5.15	C_15_H_18_O_9_	341.0878	0.06	M-H	glucocaffeic acid	[a]	Phenolic glycosides
21	5.21	C_21_H_22_O_11_	449.1099	2.18	M-H	astilbin	[a]; [b]	Flavonoid
22	5.30	C_21_H_20_O_12_	463.0888	1.38	M-H	myricitrin	[a]; [b]	Flavonoid
23	5.47	C_16_H_20_O_9_	337.0930	0.37	M-H	gentiopicroside	[a]	Other
24	5.47	C_9_H_8_O_3_	163.0398	−1.76	M-H	coumaric acid	[a]; [b]	Phenolic acid
25	5.59	C_15_H_10_O_6_	287.0549	−0.56	M+H	kaempferol	[a]; [b]	Flavonoid
26	5.76	C_16_H_18_O_9_	353.0877	−0.42	M-H	chlorogenic acid	[a]; [b]	Phenolic acid
27	5.81	C_9_H_10_O_3_	165.0555	−1.30	M-H	4-hydroxyphenyl-2-propionic acid	[a]; [b]	Phenolic acid
28	5.81	C_9_H_10_O_3_	167.0702	−1.68	M+H	ethylparaben	[a]; [b]	p-hydroxybenzoic acid ethyl ester
29	5.88	C_7_H_6_O_3_	137.0241	−2.14	M-H	3-hydroxybenzoic acid	[a]; [b]	Phenolic acid
30	5.90	C_10_H_12_O_4_	177.0562	2.26	M-H	xanthoxylin	[a]	Phenolic ketone
31	6.10	C_15_H_18_O_8_	325.0929	−0.01	M-H	coumaric acid 2-glucoside isomer	[a]; [b]	Phenolic glycosides
32	6.23	C_16_H_20_O_9_	355.1035	0.20	M-H	ferulic acid 4-glucoside isomer	[a]	Phenolic glycosides
33	6.25	C_28_H_28_N_4_O_6_S	547.1661	0.72	M-H	1-((2-methoxy-4-(((phenylsulfonyl)amino)carbonyl)phenyl)methyl)-1H-indazol-6-yl)carbamic	[a]	Herbicide
34	6.55	C_15_H_14_O_6_	289.0723	1.76	M-H	catechin	[a]; [b]	Flavonoid
35	6.55	C_13_H_16_O_9_	315.0737	4.99	M-H	protocatechuic acid 4-glucoside	[a]	Phenolic glycosides
36	6.69	C_16_H_18_O_8_	337.0929	0.02	M-H	3-p-coumaroylquinic acid	[a]; [b]	Phenolic acid
37	6.69	C_9_H_10_O_5_	197.0458	1.25	M-H	syringic acid	[a]; [b]	Phenolic acid
38	6.76	C_15_H_22_O_5_	281.1398	1.32	M-H	dihydrophasic acid	[a]; [b]	Other
39	6.93	C_41_H_28_O_26_	935.0794	0.99	M-H	casuarinin	[a]	Hydrolyzable tannin
40	7.06	C_10_H_10_O_3_	177.0557	−2.84	M-H	(S)-Isosclerone	[a]	Other
41	7.06	C_10_H_8_O_3_	175.0397	−1.85	M-H	7-hydroxy-methyl coumarin	[a]; [b]	Phenolic acid
42	7.18	C_21_H_20_O_13_	479.0824	−1.43	M-H	myricetin-3-glucoside	[a]	Phenolic glycosides
43	7.44	C_20_H_20_O_11_	435.0928	−1.10	M-H	taxifolin 3-arabinoside	[a]	Flavonoid
44	7.61	C_9_H_6_O_3_	163.0388	−1.07	M+H	3-hydroxycoumarin	[a]; [b]	Other
45	7.83	C_10_H_6_O_3_	173.0251	3.79	M-H	juglone	[a]	Quinone
46	7.92	C_23_H_22_O_12_	489.1054	3.13	M-H	quercetin 3-O-acetyl-rhamnoside	[a]; [b]	Flavonoid
47	7.95	C_21_H_24_O_11_	433.1148	1.69	M-H	catechin 3-glucoside	[a]	Phenolic glycosides
48	8.02	C_21_H_24_O_24_	435.1297	0.04	M-H	florizin	[a]; [b]	Glycoside
49	8.06	C_21_H_20_O_12_	463.0880	−0.47	M-H	quercetin 3-galactoside	[a]	Flavonoid
50	8.06	C_14_H_6_O_8_	300.9991	0.35	M-H	ellagic acid	[a]; [b]	Phenolic acid
51	8.46	C_21_H_22_O_11_	449.1092	0.49	M-H	astilbin	[a]; [b]	Flavonoid
52	8.46	C_10_H_10_O_4_	193.0516	4.91	M-H	cis-ferulic acid	[a]; [b]	Phenolic acid
53	8.51	C_20_H_18_O_11_	433.0773	−0.84	M-H	quercetin 3-xyloside	[a]	Flavonoid
54	8.64	C_10_H_12_O	149.0961	−0.15	M+H	cuminaldehyde	[a]; [b]	Aldehído aromático
55	8.66	C_21_H_20_O_11_	447.0934	0.20	M-H	quercitrin	[a]; [b]	Flavonoid
56	9.68	C_9_H_16_O_4_	187.0978	0.89	M-H	azelaic acid	[a]; [b]	Other
57	9.95	C_11_H_12_O_5_	225.0766	4.81	M+H	sinapic acid	[a]; [b]	Phenolic acid
58	11.58	C_15_H_10_O_7_	301.0356	0.75	M-H	quercetin	[a]; [b]	Flavonoid
59	13.77	C_14_H_10_O_8_	287.0207	3.06	M-H	2-(3,4-dihydroxybenzoyloxy)-4,6-dihydroxybenzoate	[a]	Phenolic compounds
60	16.69	C_15_H_10_O_6_	285.0416	3.97	M-H	luteolin	[a]; [b]	Flavonoid
61	18.83	C_18_H_12_Cl_2_N_2_O	341.0261	2.19	M-H	boscalida	[a]	Fungicide
62	28.23	C_10_H_8_O_2_	161.0599	0.91	M+H	naphthalen diol isomer	[a]; [b]	Quinone
63	31.59	C_20_H_26_NO_3_^+^	309.1744	3.10	M-H	8-O-Methyloblongin	[a]	Isoquinoline
64	31.86	C_21_H_22_O_12_	465.1037	−0.28	M-H	(-)-epicatechin 3′-O-glucuronide	[a]	Flavonoid

[a] Data basis: Progenesis QI v2.3 software; [b] Sheng et al. [38].

**Table 3:** Physicochemical parameters of the powder additive obtained via freeze-drying with different concentrations of chickpea protein as the encapsulating agent.

Analysis	Chickpea Protein Concentration (% *w*/*v*)		
	5	7.5	10
Encapsulation efficiency (%)	44 ± 3 ^a^	60 ± 8 ^b^	42 ± 5 ^a^
a_W_	0.17 ± 0.04 ^a^	0.200 ± 0.001 ^a^	0.197 ± 0.001 ^a^
Moisture (% dry basis)	7.80 ± 0.003 ^a^	6.49 ± 0.004 ^b^	6.03 ± 0.003 ^b^
Parameter L*	76.82 ± 3.42 ^a^	78.12 ± 0.07 ^a^	77.12 ± 2.74 ^a^
Parameter a*	1.7 ± 0.1 ^a^	5.9 ± 0.5 ^b^	2.78 ± 0.4 ^a^
Parameter b*	5.34 ± 0.5 ^a^	6.04 ± 5.1 ^a^	10.6 ± 0.6 ^c^
Images	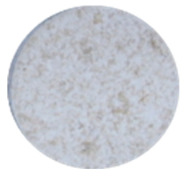	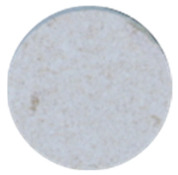	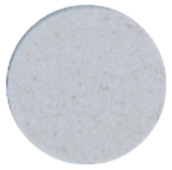

Different letters (a, b, c) indicate significant differences (*p* < 0.05) between samples.

## Data Availability

Data are contained within the article.
